# (*S*)-(+)-*cis*-4′-Benzyl­oxypraziquantel

**DOI:** 10.1107/S1600536813031735

**Published:** 2013-11-30

**Authors:** Alberto Cedillo-Cruz, María Isabel Aguilar, Helgi Jung-Cook

**Affiliations:** a Departamento de Farmacia, Facultad de Química, Universidad Nacional Autónoma de México, 04510 México, DF, Mexico

## Abstract

The asymmetric unit of the title compound, C_26_H_30_N_2_O_3_ {systematic name (*S*)-(+)-2-[*cis*-4-(benz­yloxy)cyclo­hexa­ne­carb­on­yl]-1,2,3,6,7,11b-hexa­hydro-4*H*-pyrazino­[2,1-*a*]isoquin­olin-4-one}, consists of two independent mol­ecules in which the O= C_amide_ group is *syn* to the N—C(C=O_lactam_) moiety, making dihedral angles of 2.0 (8) and 3.7 (8)°. The conformation of the 1,4-disubstituted cyclo­hexane ring is *cis* in each independent mol­ecule, with the carbonyl group occupying an equatorial position and the benz­yloxy group an axial position. In one mol­ecule, two C and one O atom of the benz­yloxy group are disordered over two sets of sites, with a refined occupancy ratio of 0.772 (8):0.228 (8). In the crystal, mol­ecules are linked by C—H⋯O inter­actions, forming ribbons parallel to the *b-*axis direction.

## Related literature

For pyrazinoisoquinolone derivatives with anthelmintic activity, see: Staudt *et al.* (1992 ▶); Jung *et al.* (2008 ▶); Thétiot-Laurent *et al.* (2013 ▶); Duan *et al.* (2012 ▶); Patra *et al.* (2013 ▶); Wang *et al.* (2013 ▶); Meier & Blaschke (2001 ▶).
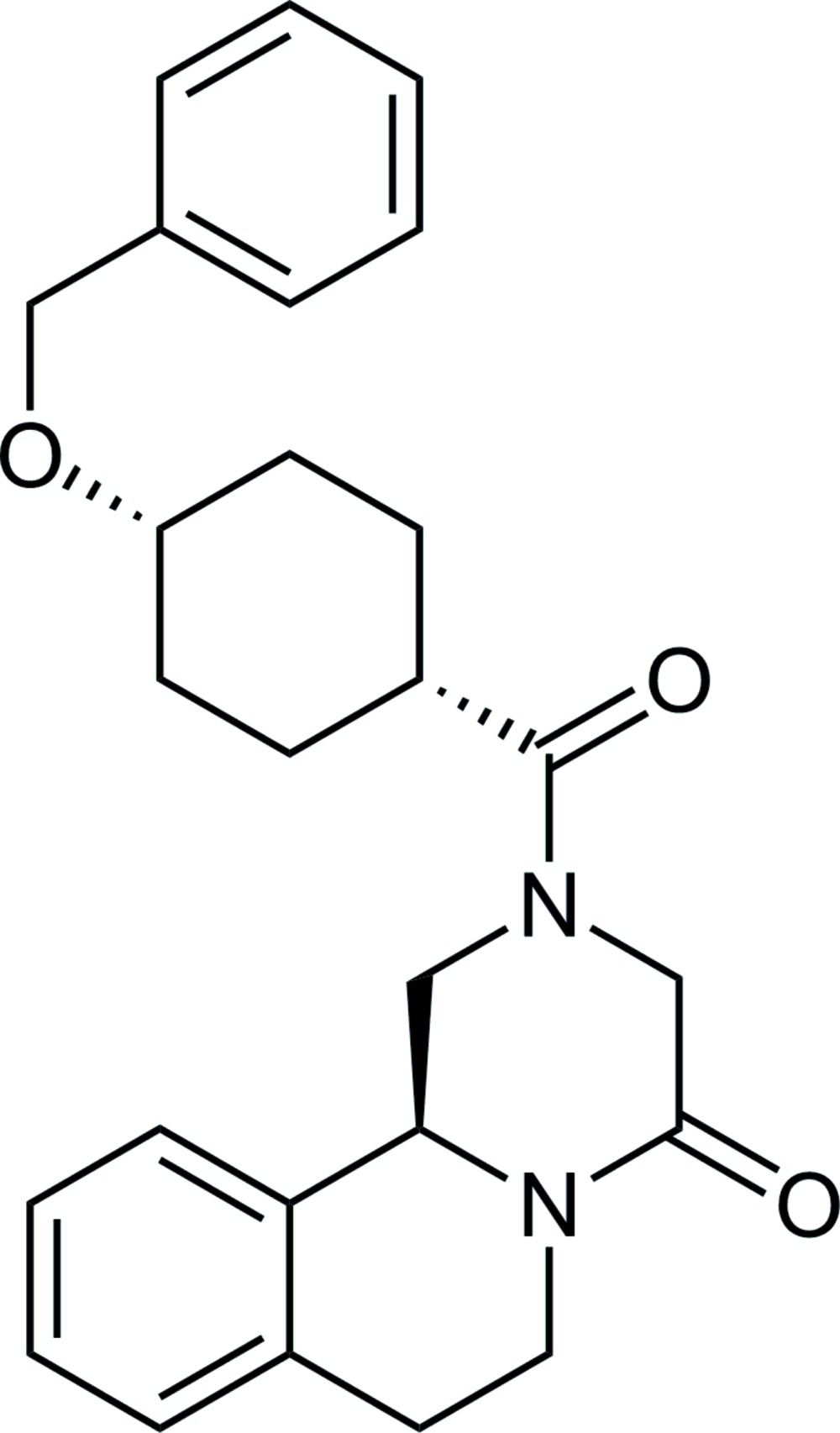


## Experimental

### 

#### Crystal data

C_26_H_30_N_2_O_3_
*M* *_r_* = 418.52
Monoclinic, 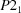

*a* = 15.007 (2) Å
*b* = 10.3322 (8) Å
*c* = 16.019 (2) Å
β = 117.399 (13)°
*V* = 2205.2 (5) Å^3^
*Z* = 4
Cu *K*α radiation
μ = 0.66 mm^−1^
*T* = 130 K
0.59 × 0.31 × 0.13 mm

#### Data collection

Oxford Diffraction Xcalibur (Atlas, Gemini) diffractometer
Absorption correction: analytical [*CrysAlis PRO* (Agilent, 2011 ▶), based on expressions derived by Clark & Reid (1995 ▶)] *T*_min_ = 0.937, *T*_max_ = 0.979
8420 measured reflections
5852 independent reflections
3845 reflections with *I* > 2σ(*I*)
*R*_int_ = 0.057

#### Refinement

*R*[*F* ^2^ > 2σ(*F* ^2^)] = 0.052
*wR*(*F* ^2^) = 0.137
*S* = 1.03
5852 reflections
558 parameters
4 restraints
H-atom parameters constrained
Δρ_max_ = 0.25 e Å^−3^
Δρ_min_ = −0.23 e Å^−3^
Absolute structure: Flack parameter determined using 868 quotients [(*I* ^+^)−(*I* ^−^)]/[(*I* ^+^)+(*I* ^−^)] (Parsons *et al.*, 2013 ▶)
Absolute structure parameter: 0.4 (3)

### 

Data collection: *CrysAlis PRO* (Agilent, 2011 ▶); cell refinement: *CrysAlis PRO*; data reduction: *CrysAlis PRO*; program(s) used to solve structure: *SHELXS2013* (Sheldrick, 2008 ▶); program(s) used to refine structure: *SHELXL2013* (Sheldrick, 2008 ▶); molecular graphics: *Mercury* (Macrae *et al.*, 2006 ▶) and *ORTEP-3 for Windows* (Farrugia, 2012 ▶); software used to prepare material for publication: *WinGX* (Farrugia, 2012 ▶) and *publCIF* (Westrip, 2010 ▶).

## Supplementary Material

Crystal structure: contains datablock(s) I, global. DOI: 10.1107/S1600536813031735/rz5093sup1.cif

Structure factors: contains datablock(s) I. DOI: 10.1107/S1600536813031735/rz5093Isup2.hkl

Additional supplementary materials:  crystallographic information; 3D view; checkCIF report

## Figures and Tables

**Table 1:** Hydrogen-bond geometry (Å, °)

*D*—H⋯*A*	*D*—H	H⋯*A*	*D*⋯*A*	*D*—H⋯*A*
C20—H20*B*⋯O2^i^	0.99	2.45	3.332 (7)	149
C20*A*—H20*C*⋯O2*A* ^ii^	0.99	2.60	3.567 (10)	165
C20*B*—H20*F*⋯O2*A* ^ii^	0.99	2.26	3.19 (3)	156

Symmetry codes: (i) 
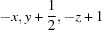
; (ii) 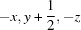.

**Fig. 1.**
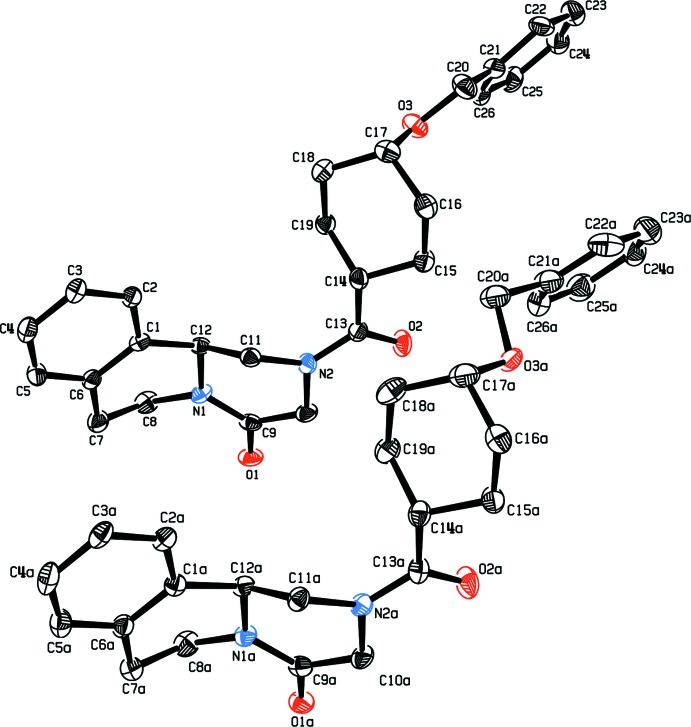
The asymmetric unit of the title compound with displacement ellipsoids drawn at the 50% probability level. Hydrogen atoms are omitted for clarity. Only the major component of the disordered atoms is shown.

**Fig. 2.**
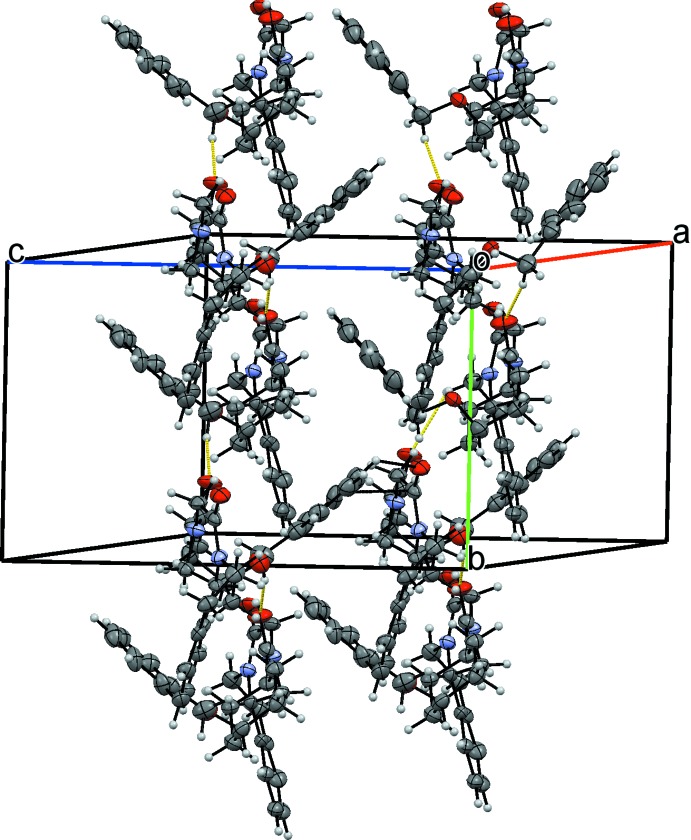
Intermolecular C—H···O interactions (dotted lines) forming ribbons parallel to the b axis in the title compound. Only the major components of the disordered atoms are shown.

## supplementary crystallographic information

### 1. Comment

The importance of pyrazinoisoquinolone derivatives as anthelmintics is well established (Jung *et al.*, 2008; Thétiot-Laurent *et al.*, 2013). Extensive number of derivatives has been synthesized, nevertheless at the date there are none with an activity comparable to that of praziquantel (Duan *et al.*, 2012; Patra *et al.*, 2013; Wang *et al.*, 2013). Such compounds usually have an asymmetric carbon atom and only one of the enantiomers presents anthelmintic activity, often the enantiomer with (*R*)-(-)-configuration (Staudt *et al.*, 1992). Herein, we report the synthesis and crystal structure of the title compound, (*S*)-(+)-*cis*-4 -benzyloxypraziquantel (Fig. 1), as a key intermediate for the synthesis of (*S*)-(+)-*cis*-4 -hydroxypraziquantel, one of the main metabolites of praziquantel (Meier & Blaschke, 2001).

The most stable conformation for (*S*)-(+)-*cis*-4'-benzyloxypraziquantel is observed where the O═ C_amide_ group is *syn* to the N–C(C═O_lactam_) moiety with a dihedral angle of 2.0 (8) and 3.7 (8)° for the two independent molecules per asymmetric unit. Furthermore, the conformation of the 1,4-disubstituted cyclohexane is *cis*, where the carbonyl moiety occupies an equatorial position and the benzyloxy moiety occupies an axial position. In the crystal (Fig. 2), molecules are linked by intermolecular C—H···O hydrogen interactions (Table 1) to form ribbons parallel to the *b* axis.

### 2. Experimental

To a solution of (*S*)-(+)-praziquanamine (505.5 mg, 2.50 mmol) and 4-methylmorpholine (1.4 ml, 12.73) in dichloromethane (35 ml) under nitrogen atmosphere, a solution of *cis*-4-(benzyloxy)cyclohexanecarbonyl chloride (586 mg, 2.50 mmol) in dichloromethane (15 ml) was added dropwise at 0°C. After stirring at room temperature for 2 h, dichloromethane (50 ml) was added. The organic layer was successively washed with 1 *M* HCl (3 · 50 ml), saturated solution of NaHCO_3_ (3 · 50 ml), water (3 · 50 ml) and dried over Na_2_SO_4_, filtered and concentrated. The residue was purified by column chromatography (SiO_2_, ethyl acetate 100%). A white powder was obtained (935.7 mg, 89% yield). m.p. 405–408 K. Single-crystals suitable for X-ray diffraction were prepared by evaporation of a solution of the title compound in methanol at room temperature.

### 3. Refinement

All H atoms were geometrically positioned and refined using a riding model, with C–H = 0.95 Å for aromatic CH, 0.99 Å for secondary CH_2_, 1.00 Å for tertiary CH, respectively, and with *U*_iso_(H) = 1.2*U*_eq_(C). The absolute configuration was not established by anomalous dispersion effects, nevertheless the absolute configuration could be assigned from the known configuration of the chiral centers. In one molecule the C20A, C21A and O3A atoms of the benzyloxy group are disordered over two sets of sites with refined site occupancies of 0.772 (8):0.228 (8). During the refinement, the C21B–C26A, C21B–C22A and C21B–C20B bond lengths were constrained to be 1.38 (1), 1.38 (2) and 1.50 (1) Å, respectively. In the last cycles of refinement, 14 outliers were omitted.

### Crystal data

**Table:**

C_26_H_30_N_2_O_3_	*D*_x_ = 1.261 Mg m^−^^3^
*M**_r_* = 418.52	Melting point = 405–408 K
Monoclinic, *P*2_1_	Cu *K*α radiation, λ = 1.54184 Å
*a* = 15.007 (2) Å	Cell parameters from 1613 reflections
*b* = 10.3322 (8) Å	θ = 3.3–73.3°
*c* = 16.019 (2) Å	µ = 0.66 mm^−^^1^
β = 117.399 (13)°	*T* = 130 K
*V* = 2205.2 (5) Å^3^	Needle, colourless
*Z* = 4	0.59 × 0.31 × 0.13 mm
*F*(000) = 896	

### Data collection

**Table:**

Oxford Diffraction Xcalibur (Atlas, Gemini) diffractometer	5852 independent reflections
Radiation source: Enhance (Cu) X-ray Source	3845 reflections with *I* > 2σ(*I*)
Graphite monochromator	*R*_int_ = 0.057
Detector resolution: 10.4685 pixels mm^-1^	θ_max_ = 68.3°, θ_min_ = 5.5°
ω scans	*h* = −17→18
Absorption correction: analytical [*CrysAlis PRO* (Agilent, 2011), based on expressions derived by Clark & Reid (1995)]	*k* = −12→7
*T*_min_ = 0.937, *T*_max_ = 0.979	*l* = −19→18
8420 measured reflections	

### Refinement

**Table:**

Refinement on *F*^2^	Hydrogen site location: inferred from neighbouring sites
Least-squares matrix: full	H-atom parameters constrained
*R*[*F*^2^ > 2σ(*F*^2^)] = 0.052	*w* = 1/[σ^2^(*F*_o_^2^) + (0.0462*P*)^2^ + 0.6847*P*] where *P* = (*F*_o_^2^ + 2*F*_c_^2^)/3
*wR*(*F*^2^) = 0.137	(Δ/σ)_max_ < 0.001
*S* = 1.03	Δρ_max_ = 0.25 e Å^−^^3^
5852 reflections	Δρ_min_ = −0.23 e Å^−^^3^
558 parameters	Extinction correction: *SHELXL2013* (Sheldrick, 2008), Fc^*^=kFc[1+0.001xFc^2^λ^3^/sin(2θ)]^-1/4^
4 restraints	Extinction coefficient: 0.00115 (17)
Primary atom site location: structure-invariant direct methods	Absolute structure: Flack parameter determined using 868 quotients [(*I*^+^)-(*I*^-^)]/[(*I*^+^)+(*I*^-^)] (Parsons *et al.*, 2013)
Secondary atom site location: difference Fourier map	Absolute structure parameter: 0.382 (265)

### Special details

**Table:**

Geometry. All e.s.d.'s (except the e.s.d. in the dihedral angle between two l.s. planes) are estimated using the full covariance matrix. The cell e.s.d.'s are taken into account individually in the estimation of e.s.d.'s in distances, angles and torsion angles; correlations between e.s.d.'s in cell parameters are only used when they are defined by crystal symmetry. An approximate (isotropic) treatment of cell e.s.d.'s is used for estimating e.s.d.'s involving l.s. planes.
Refinement. Refinement of *F*^2^ against ALL reflections. The weighted *R*-factor *wR* and goodness of fit *S* are based on *F*^2^, conventional *R*-factors *R* are based on *F*, with *F* set to zero for negative *F*^2^. The threshold expression of *F*^2^ > 2σ(*F*^2^) is used only for calculating *R*-factors(gt) *etc*. and is not relevant to the choice of reflections for refinement. *R*-factors based on *F*^2^ are statistically about twice as large as those based on *F*, and *R*-factors based on ALL data will be even larger.

### Fractional atomic coordinates and isotropic or equivalent isotropic displacement parameters (Å^2^)

**Table:**

	*x*	*y*	*z*	*U*_iso_*/*U*_eq_	Occ. (<1)
O1	0.6375 (3)	0.2009 (4)	0.7391 (3)	0.0452 (10)	
O2	0.2778 (3)	0.1973 (4)	0.5624 (3)	0.0489 (11)	
N1	0.5909 (3)	0.4128 (4)	0.7198 (3)	0.0346 (11)	
N2	0.3976 (3)	0.3440 (4)	0.5860 (3)	0.0350 (11)	
C12	0.5135 (3)	0.5133 (5)	0.6799 (3)	0.0289 (12)	
H12	0.4792	0.5224	0.7203	0.035*	
C13	0.3000 (4)	0.3110 (5)	0.5562 (4)	0.0341 (13)	
C16	0.0377 (4)	0.4631 (6)	0.4195 (4)	0.0423 (15)	
H16C	−0.0294	0.4226	0.3863	0.051*	
H16D	0.0492	0.5139	0.3729	0.051*	
C2	0.4924 (4)	0.7477 (5)	0.6361 (3)	0.0331 (13)	
H2	0.4226	0.7356	0.6154	0.040*	
C19	0.2203 (4)	0.4976 (5)	0.5981 (4)	0.0351 (13)	
H19A	0.2028	0.4424	0.6389	0.042*	
H19B	0.2880	0.5341	0.6369	0.042*	
C9	0.5738 (4)	0.2851 (6)	0.6990 (4)	0.0349 (13)	
O3	0.0086 (3)	0.4888 (4)	0.5570 (3)	0.0477 (11)	
C1	0.5582 (4)	0.6432 (5)	0.6747 (3)	0.0286 (12)	
C11	0.4367 (4)	0.4709 (5)	0.5816 (4)	0.0330 (12)	
H11A	0.3811	0.5343	0.5554	0.040*	
H11B	0.4687	0.4678	0.5396	0.040*	
C10	0.4747 (4)	0.2445 (5)	0.6220 (4)	0.0386 (14)	
H10A	0.4852	0.2116	0.5692	0.046*	
H10B	0.4494	0.1715	0.6452	0.046*	
C14	0.2207 (4)	0.4154 (5)	0.5181 (4)	0.0350 (13)	
H14	0.2362	0.4734	0.4765	0.042*	
C7	0.7334 (4)	0.5547 (6)	0.7575 (4)	0.0434 (15)	
H7A	0.7946	0.5911	0.8099	0.052*	
H7B	0.7533	0.5123	0.7132	0.052*	
C15	0.1172 (4)	0.3573 (6)	0.4602 (4)	0.0396 (14)	
H15C	0.1008	0.2991	0.5003	0.048*	
H15D	0.1172	0.3051	0.4083	0.048*	
C17	0.0396 (4)	0.5545 (6)	0.4957 (4)	0.0409 (14)	
H17	−0.0072	0.6283	0.4648	0.049*	
C6	0.6612 (4)	0.6616 (5)	0.7082 (3)	0.0324 (13)	
C22	−0.2257 (4)	0.3782 (7)	0.5544 (4)	0.0466 (16)	
H22	−0.2742	0.4268	0.5034	0.056*	
C18	0.1439 (4)	0.6076 (6)	0.5572 (4)	0.0421 (15)	
H18A	0.1432	0.6589	0.6091	0.051*	
H18B	0.1638	0.6656	0.5195	0.051*	
C8	0.6880 (4)	0.4558 (6)	0.7955 (4)	0.0444 (16)	
H8A	0.7338	0.3809	0.8208	0.053*	
H8B	0.6781	0.4941	0.8473	0.053*	
C5	0.6944 (4)	0.7833 (6)	0.6972 (4)	0.0432 (15)	
H5	0.7638	0.7960	0.7165	0.052*	
C21	−0.1238 (4)	0.3933 (6)	0.5797 (4)	0.0406 (14)	
C3	0.5275 (4)	0.8674 (5)	0.6280 (3)	0.0372 (14)	
H3	0.4820	0.9372	0.6010	0.045*	
C23	−0.2566 (5)	0.2938 (7)	0.6023 (4)	0.0524 (18)	
H23	−0.3262	0.2839	0.5838	0.063*	
C20	−0.0961 (4)	0.4757 (7)	0.5179 (5)	0.0506 (16)	
H20A	−0.1224	0.4356	0.4549	0.061*	
H20B	−0.1271	0.5623	0.5104	0.061*	
C26	−0.0555 (4)	0.3261 (6)	0.6553 (4)	0.0446 (15)	
H26	0.0142	0.3372	0.6747	0.054*	
C25	−0.0867 (4)	0.2407 (7)	0.7045 (4)	0.0514 (17)	
H25	−0.0380	0.1946	0.7569	0.062*	
C4	0.6288 (4)	0.8862 (6)	0.6590 (4)	0.0433 (15)	
H4	0.6535	0.9691	0.6542	0.052*	
C24	−0.1862 (4)	0.2229 (7)	0.6780 (4)	0.0547 (18)	
H24	−0.2072	0.1632	0.7106	0.066*	
C15A	0.0948 (4)	0.3602 (6)	−0.0635 (4)	0.0425 (14)	
H15A	0.0977	0.3212	−0.1187	0.051*	
H15B	0.0790	0.2905	−0.0300	0.051*	
C16A	0.0117 (4)	0.4614 (6)	−0.0971 (4)	0.0434 (15)	
H16A	0.0228	0.5251	−0.1375	0.052*	
H16B	−0.0537	0.4187	−0.1353	0.052*	
N1A	0.5699 (3)	0.4053 (4)	0.2033 (3)	0.0333 (10)	
O1A	0.6124 (3)	0.1921 (4)	0.2158 (3)	0.0442 (10)	
N2A	0.3746 (3)	0.3446 (4)	0.0686 (3)	0.0354 (11)	
C13A	0.2762 (4)	0.3121 (6)	0.0364 (4)	0.0389 (14)	
C1A	0.5421 (4)	0.6375 (5)	0.1644 (3)	0.0313 (12)	
O2A	0.2541 (3)	0.1984 (4)	0.0393 (3)	0.0564 (12)	
C10A	0.4491 (4)	0.2433 (5)	0.0997 (4)	0.0392 (14)	
H10C	0.4583	0.2142	0.0453	0.047*	
H10D	0.4228	0.1688	0.1205	0.047*	
C11A	0.4143 (4)	0.4725 (5)	0.0676 (4)	0.0350 (13)	
H11C	0.3594	0.5369	0.0457	0.042*	
H11D	0.4437	0.4735	0.0235	0.042*	
C9A	0.5500 (4)	0.2787 (5)	0.1779 (4)	0.0357 (13)	
C12A	0.4943 (4)	0.5080 (5)	0.1658 (4)	0.0316 (12)	
H12A	0.4615	0.5162	0.2075	0.038*	
C14A	0.1972 (4)	0.4180 (6)	0.0019 (4)	0.0387 (13)	
H14A	0.2153	0.4826	−0.0343	0.046*	
C19A	0.1934 (4)	0.4876 (6)	0.0849 (4)	0.0428 (14)	
H19C	0.1809	0.4239	0.1244	0.051*	
H19D	0.2587	0.5300	0.1241	0.051*	
C6A	0.6451 (4)	0.6533 (6)	0.1976 (4)	0.0359 (13)	
C2A	0.4790 (4)	0.7449 (5)	0.1282 (3)	0.0356 (13)	
H2A	0.4087	0.7350	0.1058	0.043*	
C5A	0.6826 (4)	0.7736 (6)	0.1926 (4)	0.0435 (15)	
H5A	0.7528	0.7837	0.2143	0.052*	
C4A	0.6206 (5)	0.8790 (6)	0.1570 (4)	0.0500 (17)	
H4A	0.6478	0.9610	0.1544	0.060*	
C8A	0.6671 (4)	0.4424 (6)	0.2795 (4)	0.0460 (16)	
H8D	0.7109	0.3653	0.3025	0.055*	
H8C	0.6581	0.4783	0.3325	0.055*	
C7A	0.7157 (4)	0.5424 (6)	0.2450 (4)	0.0443 (15)	
H7C	0.7772	0.5757	0.2988	0.053*	
H7D	0.7355	0.5017	0.2001	0.053*	
C18A	0.1100 (5)	0.5890 (6)	0.0485 (4)	0.0485 (16)	
H18C	0.1071	0.6313	0.1026	0.058*	
H18D	0.1261	0.6563	0.0135	0.058*	
C21A	−0.1020 (7)	0.3739 (16)	0.1231 (8)	0.0531 (12)	0.772 (8)
C17A	0.0086 (5)	0.5313 (6)	−0.0152 (5)	0.0531 (12)	
H17A	−0.0413	0.6033	−0.0408	0.064*	
C3A	0.5177 (4)	0.8653 (6)	0.1249 (3)	0.0398 (14)	
H3A	0.4744	0.9379	0.1008	0.048*	
C26A	−0.0429 (5)	0.2988 (7)	0.1947 (5)	0.0605 (19)	
H26A	0.0269	0.3168	0.2272	0.073*	
C23A	−0.2459 (5)	0.2612 (8)	0.1121 (5)	0.064 (2)	
H23A	−0.3163	0.2473	0.0829	0.077*	
C24A	−0.1851 (5)	0.1881 (8)	0.1876 (5)	0.0590 (19)	
H24A	−0.2136	0.1248	0.2113	0.071*	
C25A	−0.0821 (5)	0.2051 (7)	0.2303 (5)	0.0610 (19)	
H25A	−0.0394	0.1537	0.2827	0.073*	
C22A	−0.2069 (5)	0.3544 (8)	0.0777 (5)	0.064 (2)	
H22A	−0.2511	0.4107	0.0296	0.076*	
O3A	−0.0272 (4)	0.4294 (5)	0.0256 (4)	0.0387 (13)	0.772 (8)
C20A	−0.0532 (7)	0.4814 (9)	0.0929 (7)	0.0531 (12)	0.772 (8)
H20C	−0.1006	0.5544	0.0654	0.064*	0.772 (8)
H20D	0.0075	0.5139	0.1479	0.064*	0.772 (8)
O3B	0.0059 (15)	0.481 (2)	0.0661 (14)	0.0387 (13)	0.228 (8)
C20B	−0.106 (2)	0.457 (3)	0.0344 (19)	0.0531 (12)	0.228 (8)
H20E	−0.1384	0.4129	−0.0276	0.064*	0.228 (8)
H20F	−0.1422	0.5381	0.0309	0.064*	0.228 (8)
C21B	−0.1030 (16)	0.369 (5)	0.112 (3)	0.0531 (12)	0.228 (8)

### Atomic displacement parameters (Å^2^)

**Table:**

	*U*^11^	*U*^22^	*U*^33^	*U*^12^	*U*^13^	*U*^23^
O1	0.049 (2)	0.033 (2)	0.063 (3)	0.014 (2)	0.034 (2)	0.006 (2)
O2	0.043 (2)	0.028 (2)	0.069 (3)	−0.0077 (19)	0.020 (2)	0.003 (2)
N1	0.041 (2)	0.028 (3)	0.032 (2)	0.007 (2)	0.0143 (19)	−0.001 (2)
N2	0.036 (2)	0.025 (3)	0.046 (3)	−0.002 (2)	0.021 (2)	0.003 (2)
C12	0.032 (3)	0.027 (3)	0.032 (3)	0.008 (2)	0.018 (2)	0.006 (2)
C13	0.038 (3)	0.032 (3)	0.034 (3)	−0.004 (3)	0.018 (2)	−0.003 (3)
C16	0.038 (3)	0.047 (4)	0.037 (3)	−0.001 (3)	0.014 (2)	0.003 (3)
C2	0.036 (3)	0.031 (3)	0.033 (3)	−0.002 (3)	0.016 (2)	−0.001 (3)
C19	0.035 (3)	0.032 (3)	0.037 (3)	−0.001 (3)	0.016 (2)	0.000 (3)
C9	0.041 (3)	0.032 (3)	0.045 (3)	0.008 (3)	0.030 (3)	0.009 (3)
O3	0.0348 (19)	0.057 (3)	0.049 (2)	0.006 (2)	0.0168 (18)	0.010 (2)
C1	0.038 (3)	0.024 (3)	0.026 (3)	0.005 (2)	0.018 (2)	0.003 (2)
C11	0.033 (3)	0.030 (3)	0.039 (3)	0.004 (2)	0.018 (2)	0.004 (3)
C10	0.048 (3)	0.023 (3)	0.052 (4)	0.005 (3)	0.030 (3)	0.001 (3)
C14	0.035 (3)	0.031 (3)	0.040 (3)	−0.005 (3)	0.018 (2)	0.000 (3)
C7	0.036 (3)	0.041 (4)	0.044 (3)	0.000 (3)	0.011 (3)	−0.008 (3)
C15	0.040 (3)	0.035 (4)	0.041 (3)	−0.003 (3)	0.016 (3)	−0.001 (3)
C17	0.042 (3)	0.039 (4)	0.044 (3)	0.009 (3)	0.022 (3)	0.014 (3)
C6	0.035 (3)	0.032 (3)	0.029 (3)	−0.002 (3)	0.013 (2)	−0.007 (2)
C22	0.036 (3)	0.065 (5)	0.044 (3)	0.007 (3)	0.022 (3)	−0.006 (3)
C18	0.048 (3)	0.035 (4)	0.042 (3)	−0.002 (3)	0.020 (3)	−0.006 (3)
C8	0.039 (3)	0.045 (4)	0.035 (3)	0.013 (3)	0.005 (2)	0.001 (3)
C5	0.041 (3)	0.043 (4)	0.048 (3)	−0.011 (3)	0.022 (3)	−0.009 (3)
C21	0.034 (3)	0.043 (4)	0.045 (3)	0.002 (3)	0.018 (3)	−0.006 (3)
C3	0.047 (3)	0.029 (3)	0.032 (3)	−0.004 (3)	0.016 (2)	−0.001 (3)
C23	0.047 (4)	0.077 (5)	0.041 (3)	−0.012 (3)	0.027 (3)	−0.011 (4)
C20	0.036 (3)	0.048 (4)	0.065 (4)	0.004 (3)	0.020 (3)	0.012 (4)
C26	0.038 (3)	0.043 (4)	0.047 (3)	−0.005 (3)	0.015 (3)	−0.004 (3)
C25	0.054 (4)	0.049 (4)	0.041 (3)	−0.004 (3)	0.013 (3)	−0.008 (3)
C4	0.052 (4)	0.037 (4)	0.041 (3)	−0.013 (3)	0.022 (3)	−0.008 (3)
C24	0.056 (4)	0.067 (5)	0.051 (4)	−0.017 (4)	0.033 (3)	−0.012 (4)
C15A	0.037 (3)	0.040 (4)	0.047 (3)	−0.006 (3)	0.017 (3)	−0.002 (3)
C16A	0.041 (3)	0.040 (4)	0.046 (3)	0.000 (3)	0.018 (3)	0.012 (3)
N1A	0.039 (2)	0.024 (3)	0.038 (2)	0.004 (2)	0.019 (2)	0.005 (2)
O1A	0.048 (2)	0.028 (2)	0.065 (3)	0.008 (2)	0.032 (2)	0.008 (2)
N2A	0.041 (3)	0.021 (2)	0.047 (3)	0.002 (2)	0.022 (2)	0.003 (2)
C13A	0.042 (3)	0.033 (4)	0.038 (3)	−0.008 (3)	0.016 (3)	−0.002 (3)
C1A	0.038 (3)	0.033 (3)	0.024 (3)	−0.003 (3)	0.016 (2)	−0.001 (2)
O2A	0.047 (2)	0.031 (3)	0.085 (3)	−0.007 (2)	0.025 (2)	0.007 (2)
C10A	0.050 (3)	0.021 (3)	0.050 (3)	0.000 (3)	0.025 (3)	−0.001 (3)
C11A	0.044 (3)	0.027 (3)	0.036 (3)	−0.004 (3)	0.020 (3)	0.000 (3)
C9A	0.047 (3)	0.028 (3)	0.044 (3)	0.003 (3)	0.031 (3)	0.009 (3)
C12A	0.043 (3)	0.023 (3)	0.036 (3)	0.003 (3)	0.025 (2)	0.004 (2)
C14A	0.042 (3)	0.030 (3)	0.042 (3)	−0.003 (3)	0.017 (3)	0.001 (3)
C19A	0.050 (3)	0.038 (4)	0.038 (3)	0.000 (3)	0.019 (3)	0.000 (3)
C6A	0.045 (3)	0.032 (3)	0.036 (3)	−0.006 (3)	0.023 (3)	−0.004 (3)
C2A	0.048 (3)	0.030 (3)	0.032 (3)	−0.001 (3)	0.021 (3)	0.000 (3)
C5A	0.048 (3)	0.047 (4)	0.034 (3)	−0.016 (3)	0.018 (3)	−0.005 (3)
C4A	0.070 (4)	0.040 (4)	0.039 (3)	−0.024 (4)	0.024 (3)	−0.009 (3)
C8A	0.047 (3)	0.041 (4)	0.044 (3)	0.006 (3)	0.016 (3)	0.002 (3)
C7A	0.038 (3)	0.043 (4)	0.052 (4)	−0.004 (3)	0.021 (3)	−0.007 (3)
C18A	0.072 (4)	0.035 (4)	0.039 (3)	0.009 (3)	0.026 (3)	0.003 (3)
C21A	0.063 (3)	0.045 (3)	0.066 (3)	0.005 (2)	0.042 (2)	−0.002 (2)
C17A	0.063 (3)	0.045 (3)	0.066 (3)	0.005 (2)	0.042 (2)	−0.002 (2)
C3A	0.063 (4)	0.025 (3)	0.034 (3)	−0.004 (3)	0.025 (3)	−0.002 (3)
C26A	0.051 (4)	0.050 (5)	0.080 (5)	−0.010 (3)	0.029 (4)	−0.021 (4)
C23A	0.049 (4)	0.099 (6)	0.048 (4)	−0.012 (4)	0.025 (3)	−0.022 (4)
C24A	0.062 (4)	0.072 (5)	0.055 (4)	−0.027 (4)	0.037 (4)	−0.021 (4)
C25A	0.063 (4)	0.064 (5)	0.052 (4)	0.004 (4)	0.022 (3)	−0.008 (4)
C22A	0.066 (4)	0.075 (6)	0.065 (4)	0.020 (4)	0.043 (4)	0.006 (4)
O3A	0.051 (3)	0.030 (3)	0.040 (3)	−0.004 (3)	0.025 (3)	−0.009 (2)
C20A	0.063 (3)	0.045 (3)	0.066 (3)	0.005 (2)	0.042 (2)	−0.002 (2)
O3B	0.051 (3)	0.030 (3)	0.040 (3)	−0.004 (3)	0.025 (3)	−0.009 (2)
C20B	0.063 (3)	0.045 (3)	0.066 (3)	0.005 (2)	0.042 (2)	−0.002 (2)
C21B	0.063 (3)	0.045 (3)	0.066 (3)	0.005 (2)	0.042 (2)	−0.002 (2)

### Geometric parameters (Å, º)

**Table:**

O1—C9	1.232 (6)	C16A—C17A	1.517 (8)
O2—C13	1.238 (6)	C16A—H16A	0.9900
N1—C9	1.356 (7)	C16A—H16B	0.9900
N1—C12	1.467 (6)	N1A—C9A	1.362 (7)
N1—C8	1.471 (6)	N1A—C8A	1.458 (7)
N2—C13	1.358 (6)	N1A—C12A	1.465 (6)
N2—C11	1.452 (7)	O1A—C9A	1.234 (6)
N2—C10	1.455 (6)	N2A—C13A	1.364 (7)
C12—C1	1.519 (7)	N2A—C10A	1.443 (7)
C12—C11	1.526 (7)	N2A—C11A	1.452 (6)
C12—H12	1.0000	C13A—O2A	1.227 (6)
C13—C14	1.512 (7)	C13A—C14A	1.518 (8)
C16—C15	1.526 (8)	C1A—C6A	1.394 (7)
C16—C17	1.533 (8)	C1A—C2A	1.400 (7)
C16—H16C	0.9900	C1A—C12A	1.523 (7)
C16—H16D	0.9900	C10A—C9A	1.499 (7)
C2—C3	1.374 (7)	C10A—H10C	0.9900
C2—C1	1.401 (7)	C10A—H10D	0.9900
C2—H2	0.9500	C11A—C12A	1.521 (7)
C19—C18	1.531 (7)	C11A—H11C	0.9900
C19—C14	1.540 (7)	C11A—H11D	0.9900
C19—H19A	0.9900	C12A—H12A	1.0000
C19—H19B	0.9900	C14A—C19A	1.535 (8)
C9—C10	1.489 (7)	C14A—H14A	1.0000
O3—C20	1.405 (6)	C19A—C18A	1.527 (8)
O3—C17	1.436 (6)	C19A—H19C	0.9900
C1—C6	1.396 (7)	C19A—H19D	0.9900
C11—H11A	0.9900	C6A—C5A	1.382 (8)
C11—H11B	0.9900	C6A—C7A	1.507 (8)
C10—H10A	0.9900	C2A—C3A	1.385 (7)
C10—H10B	0.9900	C2A—H2A	0.9500
C14—C15	1.519 (7)	C5A—C4A	1.375 (9)
C14—H14	1.0000	C5A—H5A	0.9500
C7—C6	1.494 (7)	C4A—C3A	1.392 (8)
C7—C8	1.504 (8)	C4A—H4A	0.9500
C7—H7A	0.9900	C8A—C7A	1.509 (8)
C7—H7B	0.9900	C8A—H8D	0.9900
C15—H15C	0.9900	C8A—H8C	0.9900
C15—H15D	0.9900	C7A—H7C	0.9900
C17—C18	1.517 (8)	C7A—H7D	0.9900
C17—H17	1.0000	C18A—C17A	1.511 (9)
C6—C5	1.392 (8)	C18A—H18C	0.9900
C22—C23	1.376 (8)	C18A—H18D	0.9900
C22—C21	1.398 (7)	C21A—C26A	1.330 (12)
C22—H22	0.9500	C21A—C22A	1.412 (12)
C18—H18A	0.9900	C21A—C20A	1.526 (14)
C18—H18B	0.9900	C17A—O3B	1.418 (19)
C8—H8A	0.9900	C17A—O3A	1.466 (8)
C8—H8B	0.9900	C17A—H17A	1.0000
C5—C4	1.386 (8)	C3A—H3A	0.9500
C5—H5	0.9500	C26A—C25A	1.384 (9)
C21—C26	1.363 (8)	C26A—C21B	1.410 (12)
C21—C20	1.502 (8)	C26A—H26A	0.9500
C3—C4	1.379 (7)	C23A—C24A	1.360 (10)
C3—H3	0.9500	C23A—C22A	1.367 (10)
C23—C24	1.394 (9)	C23A—H23A	0.9500
C23—H23	0.9500	C24A—C25A	1.383 (8)
C20—H20A	0.9900	C24A—H24A	0.9500
C20—H20B	0.9900	C25A—H25A	0.9500
C26—C25	1.400 (8)	C22A—C21B	1.40 (2)
C26—H26	0.9500	C22A—H22A	0.9500
C25—C24	1.364 (8)	O3A—C20A	1.411 (10)
C25—H25	0.9500	C20A—H20C	0.9900
C4—H4	0.9500	C20A—H20D	0.9900
C24—H24	0.9500	O3B—C20B	1.54 (3)
C15A—C16A	1.523 (7)	C20B—C21B	1.523 (12)
C15A—C14A	1.528 (7)	C20B—H20E	0.9900
C15A—H15A	0.9900	C20B—H20F	0.9900
C15A—H15B	0.9900		
			
C9—N1—C12	124.0 (4)	C17A—C16A—H16A	109.3
C9—N1—C8	120.1 (4)	C15A—C16A—H16A	109.3
C12—N1—C8	115.5 (4)	C17A—C16A—H16B	109.3
C13—N2—C11	127.2 (4)	C15A—C16A—H16B	109.3
C13—N2—C10	119.6 (5)	H16A—C16A—H16B	108.0
C11—N2—C10	113.1 (4)	C9A—N1A—C8A	120.0 (5)
N1—C12—C1	111.6 (4)	C9A—N1A—C12A	123.4 (4)
N1—C12—C11	108.5 (4)	C8A—N1A—C12A	116.2 (4)
C1—C12—C11	109.9 (4)	C13A—N2A—C10A	118.9 (5)
N1—C12—H12	108.9	C13A—N2A—C11A	126.8 (5)
C1—C12—H12	108.9	C10A—N2A—C11A	114.0 (4)
C11—C12—H12	108.9	O2A—C13A—N2A	119.1 (5)
O2—C13—N2	119.8 (5)	O2A—C13A—C14A	121.6 (5)
O2—C13—C14	121.3 (5)	N2A—C13A—C14A	119.2 (5)
N2—C13—C14	118.9 (5)	C6A—C1A—C2A	119.0 (5)
C15—C16—C17	112.1 (4)	C6A—C1A—C12A	123.0 (5)
C15—C16—H16C	109.2	C2A—C1A—C12A	118.0 (4)
C17—C16—H16C	109.2	N2A—C10A—C9A	115.8 (5)
C15—C16—H16D	109.2	N2A—C10A—H10C	108.3
C17—C16—H16D	109.2	C9A—C10A—H10C	108.3
H16C—C16—H16D	107.9	N2A—C10A—H10D	108.3
C3—C2—C1	120.8 (5)	C9A—C10A—H10D	108.3
C3—C2—H2	119.6	H10C—C10A—H10D	107.4
C1—C2—H2	119.6	N2A—C11A—C12A	109.9 (4)
C18—C19—C14	110.0 (4)	N2A—C11A—H11C	109.7
C18—C19—H19A	109.7	C12A—C11A—H11C	109.7
C14—C19—H19A	109.7	N2A—C11A—H11D	109.7
C18—C19—H19B	109.7	C12A—C11A—H11D	109.7
C14—C19—H19B	109.7	H11C—C11A—H11D	108.2
H19A—C19—H19B	108.2	O1A—C9A—N1A	122.5 (5)
O1—C9—N1	123.0 (5)	O1A—C9A—C10A	118.7 (5)
O1—C9—C10	118.2 (5)	N1A—C9A—C10A	118.9 (5)
N1—C9—C10	118.8 (5)	N1A—C12A—C11A	110.1 (4)
C20—O3—C17	112.8 (4)	N1A—C12A—C1A	111.2 (4)
C6—C1—C2	119.7 (5)	C11A—C12A—C1A	110.4 (4)
C6—C1—C12	122.5 (5)	N1A—C12A—H12A	108.4
C2—C1—C12	117.7 (4)	C11A—C12A—H12A	108.4
N2—C11—C12	109.8 (4)	C1A—C12A—H12A	108.4
N2—C11—H11A	109.7	C13A—C14A—C15A	109.8 (5)
C12—C11—H11A	109.7	C13A—C14A—C19A	110.9 (5)
N2—C11—H11B	109.7	C15A—C14A—C19A	110.5 (5)
C12—C11—H11B	109.7	C13A—C14A—H14A	108.5
H11A—C11—H11B	108.2	C15A—C14A—H14A	108.5
N2—C10—C9	116.0 (5)	C19A—C14A—H14A	108.5
N2—C10—H10A	108.3	C18A—C19A—C14A	110.0 (4)
C9—C10—H10A	108.3	C18A—C19A—H19C	109.7
N2—C10—H10B	108.3	C14A—C19A—H19C	109.7
C9—C10—H10B	108.3	C18A—C19A—H19D	109.7
H10A—C10—H10B	107.4	C14A—C19A—H19D	109.7
C13—C14—C15	111.0 (5)	H19C—C19A—H19D	108.2
C13—C14—C19	111.2 (4)	C5A—C6A—C1A	119.6 (6)
C15—C14—C19	109.4 (4)	C5A—C6A—C7A	120.1 (5)
C13—C14—H14	108.4	C1A—C6A—C7A	120.2 (5)
C15—C14—H14	108.4	C3A—C2A—C1A	120.8 (5)
C19—C14—H14	108.4	C3A—C2A—H2A	119.6
C6—C7—C8	111.3 (5)	C1A—C2A—H2A	119.6
C6—C7—H7A	109.4	C4A—C5A—C6A	121.3 (6)
C8—C7—H7A	109.4	C4A—C5A—H5A	119.3
C6—C7—H7B	109.4	C6A—C5A—H5A	119.3
C8—C7—H7B	109.4	C5A—C4A—C3A	119.8 (6)
H7A—C7—H7B	108.0	C5A—C4A—H4A	120.1
C14—C15—C16	110.8 (5)	C3A—C4A—H4A	120.1
C14—C15—H15C	109.5	N1A—C8A—C7A	109.8 (5)
C16—C15—H15C	109.5	N1A—C8A—H8D	109.7
C14—C15—H15D	109.5	C7A—C8A—H8D	109.7
C16—C15—H15D	109.5	N1A—C8A—H8C	109.7
H15C—C15—H15D	108.1	C7A—C8A—H8C	109.7
O3—C17—C18	106.8 (4)	H8D—C8A—H8C	108.2
O3—C17—C16	111.1 (5)	C6A—C7A—C8A	111.0 (5)
C18—C17—C16	112.1 (5)	C6A—C7A—H7C	109.4
O3—C17—H17	108.9	C8A—C7A—H7C	109.4
C18—C17—H17	108.9	C6A—C7A—H7D	109.4
C16—C17—H17	108.9	C8A—C7A—H7D	109.4
C5—C6—C1	118.1 (5)	H7C—C7A—H7D	108.0
C5—C6—C7	121.3 (5)	C17A—C18A—C19A	112.4 (5)
C1—C6—C7	120.5 (5)	C17A—C18A—H18C	109.1
C23—C22—C21	120.9 (6)	C19A—C18A—H18C	109.1
C23—C22—H22	119.6	C17A—C18A—H18D	109.1
C21—C22—H22	119.6	C19A—C18A—H18D	109.1
C17—C18—C19	110.8 (5)	H18C—C18A—H18D	107.9
C17—C18—H18A	109.5	C26A—C21A—C22A	120.2 (10)
C19—C18—H18A	109.5	C26A—C21A—C20A	118.1 (9)
C17—C18—H18B	109.5	C22A—C21A—C20A	121.7 (8)
C19—C18—H18B	109.5	O3B—C17A—C18A	88.3 (9)
H18A—C18—H18B	108.1	O3A—C17A—C18A	116.4 (5)
N1—C8—C7	109.4 (5)	O3B—C17A—C16A	130.2 (10)
N1—C8—H8A	109.8	O3A—C17A—C16A	101.9 (5)
C7—C8—H8A	109.8	C18A—C17A—C16A	110.4 (5)
N1—C8—H8B	109.8	O3B—C17A—H17A	108.4
C7—C8—H8B	109.8	C18A—C17A—H17A	108.4
H8A—C8—H8B	108.2	C16A—C17A—H17A	108.4
C4—C5—C6	121.7 (5)	C2A—C3A—C4A	119.4 (6)
C4—C5—H5	119.1	C2A—C3A—H3A	120.3
C6—C5—H5	119.1	C4A—C3A—H3A	120.3
C26—C21—C22	118.5 (6)	C21A—C26A—C25A	121.5 (8)
C26—C21—C20	123.4 (5)	C25A—C26A—C21B	122.4 (13)
C22—C21—C20	118.0 (5)	C25A—C26A—H26A	118.8
C2—C3—C4	120.0 (5)	C21B—C26A—H26A	118.8
C2—C3—H3	120.0	C24A—C23A—C22A	120.9 (6)
C4—C3—H3	120.0	C24A—C23A—H23A	119.6
C22—C23—C24	120.2 (6)	C22A—C23A—H23A	119.6
C22—C23—H23	119.9	C23A—C24A—C25A	120.5 (7)
C24—C23—H23	119.9	C23A—C24A—H24A	119.7
O3—C20—C21	110.6 (5)	C25A—C24A—H24A	119.7
O3—C20—H20A	109.5	C24A—C25A—C26A	118.4 (7)
C21—C20—H20A	109.5	C24A—C25A—H25A	120.8
O3—C20—H20B	109.5	C26A—C25A—H25A	120.8
C21—C20—H20B	109.5	C23A—C22A—C21B	121.4 (11)
H20A—C20—H20B	108.1	C23A—C22A—C21A	118.4 (7)
C21—C26—C25	120.7 (6)	C23A—C22A—H22A	119.3
C21—C26—H26	119.6	C21B—C22A—H22A	119.3
C25—C26—H26	119.6	C20A—O3A—C17A	110.8 (6)
C24—C25—C26	120.8 (6)	O3A—C20A—C21A	107.9 (9)
C24—C25—H25	119.6	O3A—C20A—H20C	110.1
C26—C25—H25	119.6	C21A—C20A—H20C	110.1
C3—C4—C5	119.5 (5)	O3A—C20A—H20D	110.1
C3—C4—H4	120.3	C21A—C20A—H20D	110.1
C5—C4—H4	120.3	H20C—C20A—H20D	108.4
C25—C24—C23	118.9 (6)	C17A—O3B—C20B	104.2 (15)
C25—C24—H24	120.6	O3B—C20B—C21B	102 (2)
C23—C24—H24	120.6	O3B—C20B—H20E	111.3
C16A—C15A—C14A	112.1 (5)	C21B—C20B—H20E	111.3
C16A—C15A—H15A	109.2	O3B—C20B—H20F	111.3
C14A—C15A—H15A	109.2	C21B—C20B—H20F	111.3
C16A—C15A—H15B	109.2	H20E—C20B—H20F	109.2
C14A—C15A—H15B	109.2	C22A—C21B—C26A	115.4 (16)
H15A—C15A—H15B	107.9	C22A—C21B—C20B	97.6 (14)
C17A—C16A—C15A	111.6 (5)	C26A—C21B—C20B	147 (2)
			
C9—N1—C12—C1	−151.7 (5)	C12A—N1A—C9A—C10A	7.3 (8)
C8—N1—C12—C1	36.5 (6)	N2A—C10A—C9A—O1A	170.6 (5)
C9—N1—C12—C11	−30.5 (7)	N2A—C10A—C9A—N1A	−10.6 (8)
C8—N1—C12—C11	157.7 (4)	C9A—N1A—C12A—C11A	−28.3 (7)
C11—N2—C13—O2	178.1 (5)	C8A—N1A—C12A—C11A	159.1 (5)
C10—N2—C13—O2	2.0 (8)	C9A—N1A—C12A—C1A	−151.0 (5)
C11—N2—C13—C14	−3.3 (8)	C8A—N1A—C12A—C1A	36.4 (6)
C10—N2—C13—C14	−179.4 (5)	N2A—C11A—C12A—N1A	52.6 (5)
C12—N1—C9—O1	−173.6 (5)	N2A—C11A—C12A—C1A	175.8 (4)
C8—N1—C9—O1	−2.2 (9)	C6A—C1A—C12A—N1A	−1.7 (7)
C12—N1—C9—C10	8.5 (8)	C2A—C1A—C12A—N1A	178.5 (4)
C8—N1—C9—C10	179.9 (5)	C6A—C1A—C12A—C11A	−124.2 (5)
C3—C2—C1—C6	2.1 (8)	C2A—C1A—C12A—C11A	56.0 (6)
C3—C2—C1—C12	−179.0 (5)	O2A—C13A—C14A—C15A	−24.5 (8)
N1—C12—C1—C6	−2.5 (7)	N2A—C13A—C14A—C15A	158.3 (5)
C11—C12—C1—C6	−122.8 (5)	O2A—C13A—C14A—C19A	97.9 (7)
N1—C12—C1—C2	178.7 (4)	N2A—C13A—C14A—C19A	−79.2 (7)
C11—C12—C1—C2	58.4 (6)	C16A—C15A—C14A—C13A	177.6 (5)
C13—N2—C11—C12	123.1 (5)	C16A—C15A—C14A—C19A	55.0 (7)
C10—N2—C11—C12	−60.6 (6)	C13A—C14A—C19A—C18A	−177.2 (5)
N1—C12—C11—N2	55.0 (5)	C15A—C14A—C19A—C18A	−55.2 (7)
C1—C12—C11—N2	177.3 (4)	C2A—C1A—C6A—C5A	−1.2 (8)
C13—N2—C10—C9	−146.1 (5)	C12A—C1A—C6A—C5A	179.0 (5)
C11—N2—C10—C9	37.3 (7)	C2A—C1A—C6A—C7A	174.3 (5)
O1—C9—C10—N2	171.7 (5)	C12A—C1A—C6A—C7A	−5.5 (8)
N1—C9—C10—N2	−10.4 (7)	C6A—C1A—C2A—C3A	0.3 (8)
O2—C13—C14—C15	−20.1 (7)	C12A—C1A—C2A—C3A	−179.9 (5)
N2—C13—C14—C15	161.4 (5)	C1A—C6A—C5A—C4A	1.1 (9)
O2—C13—C14—C19	101.9 (6)	C7A—C6A—C5A—C4A	−174.4 (5)
N2—C13—C14—C19	−76.7 (6)	C6A—C5A—C4A—C3A	−0.2 (9)
C18—C19—C14—C13	176.5 (4)	C9A—N1A—C8A—C7A	124.0 (5)
C18—C19—C14—C15	−60.6 (6)	C12A—N1A—C8A—C7A	−63.2 (6)
C13—C14—C15—C16	−178.4 (4)	C5A—C6A—C7A—C8A	155.6 (5)
C19—C14—C15—C16	58.6 (6)	C1A—C6A—C7A—C8A	−19.8 (7)
C17—C16—C15—C14	−54.5 (6)	N1A—C8A—C7A—C6A	51.7 (6)
C20—O3—C17—C18	158.3 (5)	C14A—C19A—C18A—C17A	57.1 (7)
C20—O3—C17—C16	−79.2 (6)	C19A—C18A—C17A—O3B	76.0 (9)
C15—C16—C17—O3	−67.5 (6)	C19A—C18A—C17A—O3A	58.9 (7)
C15—C16—C17—C18	51.9 (7)	C19A—C18A—C17A—C16A	−56.7 (7)
C2—C1—C6—C5	−3.4 (8)	C15A—C16A—C17A—O3B	−51.0 (13)
C12—C1—C6—C5	177.8 (5)	C15A—C16A—C17A—O3A	−69.6 (6)
C2—C1—C6—C7	174.3 (5)	C15A—C16A—C17A—C18A	54.8 (7)
C12—C1—C6—C7	−4.5 (8)	C1A—C2A—C3A—C4A	0.6 (8)
C8—C7—C6—C5	156.6 (5)	C5A—C4A—C3A—C2A	−0.7 (8)
C8—C7—C6—C1	−21.0 (7)	C22A—C21A—C26A—C25A	−3.8 (19)
O3—C17—C18—C19	68.3 (6)	C20A—C21A—C26A—C25A	175.5 (9)
C16—C17—C18—C19	−53.6 (6)	C22A—C21A—C26A—C21B	96 (15)
C14—C19—C18—C17	58.2 (6)	C20A—C21A—C26A—C21B	−85 (14)
C9—N1—C8—C7	124.8 (5)	C22A—C23A—C24A—C25A	−1.1 (11)
C12—N1—C8—C7	−63.1 (6)	C23A—C24A—C25A—C26A	0.6 (10)
C6—C7—C8—N1	52.4 (6)	C21A—C26A—C25A—C24A	1.9 (13)
C1—C6—C5—C4	3.5 (8)	C21B—C26A—C25A—C24A	−6 (3)
C7—C6—C5—C4	−174.2 (5)	C24A—C23A—C22A—C21B	7 (3)
C23—C22—C21—C26	−2.3 (9)	C24A—C23A—C22A—C21A	−0.6 (13)
C23—C22—C21—C20	173.4 (6)	C26A—C21A—C22A—C23A	3.1 (19)
C1—C2—C3—C4	−0.8 (8)	C20A—C21A—C22A—C23A	−176.2 (10)
C21—C22—C23—C24	0.7 (10)	C26A—C21A—C22A—C21B	−113 (17)
C17—O3—C20—C21	173.5 (5)	C20A—C21A—C22A—C21B	68 (15)
C26—C21—C20—O3	−9.0 (9)	O3B—C17A—O3A—C20A	37.7 (15)
C22—C21—C20—O3	175.5 (5)	C18A—C17A—O3A—C20A	70.7 (8)
C22—C21—C26—C25	2.0 (9)	C16A—C17A—O3A—C20A	−169.1 (6)
C20—C21—C26—C25	−173.4 (6)	C17A—O3A—C20A—C21A	172.9 (6)
C21—C26—C25—C24	0.0 (9)	C26A—C21A—C20A—O3A	88.9 (14)
C2—C3—C4—C5	0.8 (8)	C22A—C21A—C20A—O3A	−91.8 (14)
C6—C5—C4—C3	−2.2 (9)	O3A—C17A—O3B—C20B	−50.1 (17)
C26—C25—C24—C23	−1.6 (10)	C18A—C17A—O3B—C20B	159.1 (16)
C22—C23—C24—C25	1.3 (10)	C16A—C17A—O3B—C20B	−85.4 (18)
C14A—C15A—C16A—C17A	−54.9 (7)	C17A—O3B—C20B—C21B	164 (3)
C10A—N2A—C13A—O2A	3.7 (8)	C23A—C22A—C21B—C26A	−12 (6)
C11A—N2A—C13A—O2A	177.6 (5)	C21A—C22A—C21B—C26A	56 (12)
C10A—N2A—C13A—C14A	−179.0 (5)	C23A—C22A—C21B—C20B	166.3 (18)
C11A—N2A—C13A—C14A	−5.1 (8)	C21A—C22A—C21B—C20B	−126 (19)
C13A—N2A—C10A—C9A	−147.8 (5)	C21A—C26A—C21B—C22A	−73 (12)
C11A—N2A—C10A—C9A	37.5 (7)	C25A—C26A—C21B—C22A	11 (6)
C13A—N2A—C11A—C12A	126.6 (5)	C21A—C26A—C21B—C20B	110 (21)
C10A—N2A—C11A—C12A	−59.2 (6)	C25A—C26A—C21B—C20B	−165 (7)
C8A—N1A—C9A—O1A	−1.6 (8)	O3B—C20B—C21B—C22A	179 (3)
C12A—N1A—C9A—O1A	−173.9 (5)	O3B—C20B—C21B—C26A	−4 (10)
C8A—N1A—C9A—C10A	179.6 (5)		

### Hydrogen-bond geometry (Å, º)

**Table:**

*D*—H···*A*	*D*—H	H···*A*	*D*···*A*	*D*—H···*A*
C20—H20*B*···O2^i^	0.99	2.45	3.332 (7)	149
C20*A*—H20*C*···O2*A*^ii^	0.99	2.60	3.567 (10)	165
C20*B*—H20*F*···O2*A*^ii^	0.99	2.26	3.19 (3)	156

Symmetry codes: (i) −*x*, *y*+1/2, −*z*+1; (ii) −*x*, *y*+1/2, −*z*.
